# Editorial: Educator burnout – Improving the understanding of challenges and advancing insights for interventions and prevention

**DOI:** 10.3389/fpsyg.2026.1911129

**Published:** 2026-07-22

**Authors:** Xiantong Yang

**Affiliations:** School of Psychology, Fujian Normal University, Fuzhou, China

**Keywords:** educator burnout, occupational stress, organizational climate, teacher wellbeing, work engagement

Occupational health and wellbeing among educators have become an increasingly urgent global concern. Across educational settings, rising levels of stress-related ill-being, burnout, and voluntary turnover have drawn attention to the sustainability of educational work and the conditions under which educators teach, lead, and support students. Reports from the United States, for example, have shown that more than 60% of higher education employees experience work-related burnout, while approximately 40% of students report clinically significant symptoms of depression. At the same time, voluntary turnover among higher education employees has increased markedly in recent years. International evidence suggests that these are not isolated national trends, but part of a broader and deeply consequential challenge affecting educator wellbeing, student learning, and the quality of educational systems worldwide.

Against this backdrop, this Research Topic, *Educator burnout – Improving the understanding of challenges and advancing insights for interventions and prevention*, was developed to advance understanding of the factors contributing to educator burnout and related occupational health challenges, while also prioritizing insights for intervention and prevention. The Topic sought to explore not only the causes and consequences of educator stress, burnout, and turnover, but also the organizational, emotional, and psychological processes through which these outcomes develop. Contributions were invited from multiple disciplinary perspectives, including work and organizational psychology, educational psychology, and health psychology, with particular interest in cross-cultural perspectives, emotional labor, coping, workplace culture, leadership, and supportive practices in educational settings. The resulting Research Topic reflects that multidisciplinary vision and offers a rich, internationally relevant portrait of educator wellbeing.

The 20 articles included in this Research Topic span preschool, primary, secondary, and higher education settings, as well as educational administration, counseling, and cross-cultural teaching contexts. They draw on data from China, Mexico, Taiwan, Portugal, Italy, Switzerland, Bulgaria, and South Asian countries, among others. Methodologically, the Research Topic includes cross-sectional surveys, longitudinal studies, qualitative interview and focus group research, structural equation modeling, cross-lagged panel models, classification tree analysis, polynomial regression, and network analysis. Taken together, these contributions demonstrate that educator burnout is best understood as a multidimensional phenomenon shaped by the interaction of job demands, emotional regulation, organizational support, leadership, career stage, and sociocultural context.

A major theme running across the Research Topic is the persistent burden of occupational stress and burnout in educational work. In “*Associated factors and rise of burnout in Mexican teachers: The impact of school principals, age, and years of teaching experience*,” Cortez Soto and Heredia Escorza showed that principal support functioned as the strongest protective factor against burnout among preschool and elementary school teachers in Mexico, whereas excessive demands, younger age, fewer years of teaching experience, grade level taught, and urban school location increased burnout risk. In “*Gender and teaching experience differences in occupational stress and psychological symptoms among Chinese junior high school teachers*,” Bian and Jiang reported no meaningful gender differences in occupational stress or psychological symptoms, but found a pronounced non-linear association with teaching experience, with teachers having over 30 years of experience showing lower stress and fewer symptoms than early- and mid-career groups. In “*Organizational health factors: multidimensional insights into occupational stress in higher education*,” Souto et al. found that work-family conflict and job demands were major risk factors for higher education teachers' health and wellbeing, whereas resilient coping and meaningfulness of work served as protective factors. Similarly, in “*Exploring mental health challenges encountered by educators in the South Asian countries—a qualitative survey*,” Kazimi et al. documented common experiences across seven South Asian countries, including burnout, excessive workload, exclusion from decision-making, exploitation, professional jealousy, and toxic workplaces, alongside cultural barriers to discussing mental health and limited access to workplace support. In “*Stress and coping among university faculty and staff at a medical university in the post-pandemic context: a qualitative analysis*,” Slavova and Tarpomanova further highlighted work overload, problematic workplace relationships, and administrative difficulties as key post-pandemic stressors for university faculty and staff.

Relatedly, several studies in the Topic focused specifically on burnout as an outcome of job demands and emotionally taxing work conditions. In “*The impact of workload and emotional demands on turnover intentions: the mediating and moderating effects of job burnout*,” Hung et al. found that workload and emotional demands contributed to preschool teachers' turnover intentions through job burnout, and that burnout also intensified the association between workload and turnover intention. In “*Untangling teacher burnout: a network analysis of demands, resources, and out-of-field teaching challenges in rural China*,” Huo showed that emotional exhaustion was the central burnout component among rural English teachers in China, with workload stress acting as the primary bridge connecting job demands and resources to burnout, while job satisfaction demonstrated strong protective associations. In “*The role of ethical climate on teachers' wellbeing: relationship with psychophysical wellbeing and general health*,” Borrelli et al. demonstrated that ethical climate in educational settings was significantly associated with lower levels of teacher stress and depression, underscoring the importance of organizational ethics as part of occupational health.

Another important cluster of articles examined emotional labor, emotional regulation, and their implications for educator wellbeing. In “*The impact of surface acting and mindfulness on preschool teachers' burnout: the roles of emotional empathy and perceived organizational support*,” He et al. reported that the interaction between surface acting and mindfulness predicted burnout in a non-linear way, with emotional empathy partly mediating this relationship and perceived organizational support buffering both direct and indirect negative effects. In “*Differential effects of emotional labor strategies on university counselors' job burnout: the dual role of psychological capital as a mediator and a moderator*,” Song and Yang extended this line of inquiry by showing that emotional labor strategies were differentially associated with counselor burnout and that psychological capital operated both as a mediating and moderating resource. In “*Novice teachers' emotional labor: a study of volunteer Chinese teachers in African Confucius Institutes*,” Wu and Yan demonstrated how novice volunteer Chinese teachers navigated emotional labor through the influence of professional, organizational, sociocultural, and personal emotional rules, revealing the complexity of emotional regulation in cross-cultural teaching contexts and the limited institutional support available to these teachers.

Beyond documenting risks, the Research Topic also highlights the importance of supportive organizational environments and leadership in promoting educator wellbeing and reducing stress. In “*How the teacher development ecosystem influences career success: the chain mediation role of school climate and job crafting*,” Xu et al. found that the teacher development ecosystem positively predicted teachers' career success and that school climate and job crafting jointly mediated this relationship. In “*Exploring the influencing effect of tertiary teachers' perceived organizational support and teacher self-efficacy on their job satisfaction: the mediating role of teacher work engagement*,” Chen showed that perceived organizational support and teacher self-efficacy were positively related to job satisfaction among tertiary teachers, with work engagement serving as a partial mediator. In “*How transformational leadership reduces teachers' role stress: dual mediation of affective commitment and job satisfaction*,” Yong and Zhang found that transformational leadership reduced role stress both directly and indirectly through affective commitment and job satisfaction. In “*How paternalistic leadership influences teachers' resistance to STEM reform: the mediating role of teachers' STEM literacy and the moderating role of work engagement*,” Zhang and Zhao reported that authoritarian leadership was positively related to teachers' resistance to STEM reform, whereas benevolent-moral leadership influenced resistance indirectly through STEM literacy, with work engagement moderating the relationship between leadership and STEM literacy. Collectively, these studies reinforce one of the Topic's core concerns: workplace culture, leadership, and institutional support are central to occupational health and cannot be treated as peripheral background factors.

A further strength of this Research Topic is its attention to personal resources, self-regulation, and psychological mechanisms that may help explain why some educators remain engaged and well while others struggle. In “*The impact of trait mindfulness on work engagement among primary and secondary school teachers: a moderated mediation model*,” Han and Zhang showed that trait mindfulness positively predicted work engagement, partly through job satisfaction, and that perceived organizational support strengthened both the direct and mediated pathways. In “*Why coaching matters: exploring the interplay of teacher self-regulation and well-being with a longitudinal multigroup model*,” Bührer et al. reported that self-regulation predicted work engagement and emotional exhaustion only among early career teachers who received training plus online coaching, suggesting that coaching may activate self-regulation as an effective resource. In “*The longitudinal impact of emotional intelligence and psychological empowerment on work engagement among university administrators: a cross-lagged panel model approach*,” Zhou and Wang found that emotional intelligence and psychological empowerment both positively predicted later work engagement among university administrators, with no significant gender differences in these longitudinal effects. These findings align closely with the Topic's emphasis on coping mechanisms, emotional regulation, and supportive strategies for intervention and prevention.

Several articles also broaden the scope of educator wellbeing by examining outcomes beyond burnout itself, including career success, job satisfaction, work engagement, and teaching effectiveness. In “*The impact of work concerns on teaching effectiveness: a structural equation modeling and multiple regression analysis in Chinese private universities*,” Liang et al. found that task concerns and impact concerns significantly influenced multiple dimensions of teacher effectiveness among young lecturers in private universities, suggesting that work concerns may reflect not only strain but also deeper professional motivation. In “*Navigating triple demands: work-life balance challenges and coping strategies among Chinese university teachers pursuing further education*,” Wu and Wu revealed how university teachers balancing professional responsibilities, personal life, and further education experienced multiple forms of strain, while also identifying coping strategies related to time management, family support, institutional policy, and mental health maintenance. These contributions are valuable because they shift the discussion from a purely deficit-based view of educator occupational health toward a broader focus on sustainable professional functioning.

Taken together, the studies in this Research Topic make several important contributions to the literature. First, they show that educator burnout is not a narrow psychological state, but a complex occupational phenomenon shaped by interactions among job demands, emotional labor, leadership, workplace climate, organizational support, and personal resources. Second, they demonstrate that the consequences of educator burnout and related strain likely extend beyond the individual educator, with implications for teaching quality, professional retention, and the educational experiences of pupils and students. Third, they offer actionable insights for intervention and prevention. Across the Research Topic, the practical implications converge around several priorities: reducing excessive workload and emotional demands; strengthening leadership and principal support; promoting ethical, supportive, and development-oriented institutional climates; offering coaching, mindfulness training, emotional regulation support, and other resource-building interventions; and tailoring support to educators' career stages, professional roles, and sociocultural contexts.

The Topic also points to several directions for future research. More longitudinal and intervention-based studies are needed to identify sustainable causal pathways and determine which support strategies produce lasting improvements in educator wellbeing. Cross-cultural comparisons should continue to be expanded, especially because some findings in this Research Topic challenge assumptions that may not generalize across contexts. Future research would also benefit from more direct attention to bidirectional relationships between educator and student wellbeing, to work rumination, detachment, and recovery processes, and to emerging issues such as digital fatigue in increasingly technologized educational environments. Multidisciplinary work remains essential for addressing the complexity of educator burnout and designing effective prevention efforts.

In conclusion, the 20 articles in this Research Topic provide a rich and timely body of evidence showing that educator burnout is both a pressing global challenge and a preventable one. By integrating insights from diverse educational sectors, professional roles, national contexts, and theoretical traditions, this Research Topic deepens understanding of the challenges educators face while also advancing the evidence base for intervention and prevention. Supporting educator well-being is not only a matter of protecting individuals from occupational harm; it is also fundamental to sustaining healthy educational organizations and improving outcomes for students. We hope this Research Topic stimulates continued research, dialogue, and action toward more supportive, humane, and sustainable educational work environments.

